# Prevalence of urinary iodine concentration among school children: in Dessie City, Ethiopia

**DOI:** 10.1186/s12887-021-02887-7

**Published:** 2021-09-24

**Authors:** Birtukan Shiferaw Ayalew, Seid Legesse Hassen, Tefera Alemu Marefiyaw, Mohammed Seid Yesuf, Daniel Dagne Abebe, Minwuyelet Maru Temesgen

**Affiliations:** 1 Research and Technology Transfer Directorate, Amhara Public Health Institute Dessie Branch, Dessie, Amhara Region Ethiopia; 2 Public Health Emergency Management Directorate, Amhara Public Health Institute, Bahir Dar, Amhara Region Ethiopia; 3 Laboratory directorates, Amhara Public Health Institute Dessie Branch, Dessie, Amhara Region Ethiopia

**Keywords:** Iodine deficiency, Urinary iodine concentration, Amhara region, Dessie, Ethiopia

## Abstract

**Background:**

Urinary iodine is recommended by the world health organization as the main indicator to assess iodine status in a population. Despite this recommendation little is known about urinary iodine concentration in the study area. Therefore, this study aimed to determine the level of urinary iodine concentration among school-aged children.

**Methods:**

An institution-based cross-sectional study design was used to assess the level of urinary iodine from April to June 2019 and a systematic random sampling technique was applied to select study participants. Socio-demographic characteristics were assessed using a pretested structured questionnaire and the laboratory method by Sandell–Kolthoff reaction method was used. Data were cleaned, coded, and entered into Epi data version 3.1 and then exported to SPSS version 21 software for analysis.

**Result:**

A total of 634 study participants were enrolled in the study with a median age of 12 years (±SD = 2.0). The majority of the children were females (55.4%) and more than half of respondents report the use of iodized salt always. Median urinary iodine concentration was 158.5 μg/L (±SD = 104.1) with minimum and maximum values of 5.1 μg/L and 528.8 μg/L, respectively. The overall iodine deficiency in this study was 18.6% and severe deficiency constituted 7.4%.

**Conclusions:**

The iodine deficiency of the school children aged 6 to 14 in the present study was 18.6% indicating high prevalence. A high proportion of iodine deficiency was observed among females and it increases as age increases. This indicates the need for an additional strategy to control iodine deficiency.

## Introduction

The proper functioning of many-body systems, including the mammary glands, thyroid glands, salivary glands, and gastric mucosa, needs a sufficient amount of iodine [1]. Especially, iodine is essential for the synthesis of thyroid hormones which are necessary for growth and development. The primary source of iodine is the intake of iodized salt and the diet such as dairy products and grains. However, excess uptake of certain food types like seaweed, nutritional supplements, and some medications, such as amiodarone can be responsible for excess iodine. The relation between iodine intake and the risk of health problems can be described by a U-shaped curve, with extremes of both high and low intakes impose adverse consequences on health [2, 3]. Iodine does not occur naturally in foods; rather, it is present in the soil and is ingested through foods grown on that soil. In recent years, depletion of the iodine content of the soil due to flooding, deforestation, and erosion increases the risk of iodine deficiency (ID) which is associated with geological conditions and socio-economic factors [4], including large families, poor economic conditions, low maternal education, poor maternal knowledge about iodized salt, and place of residence [5]. Moreover, several studies have revealed that adding salt during food preparation, use of unpacked salt, storage of salts for a longer duration and near to fire, in an open container, or exposure to heat and sunlight were associated with iodine deficiency. Purchasing salt greater than 5 kg at once, consumption of food items containing goitrogens, and co-existing micronutrient deficiencies (iron, selenium, and vitamin A) are also reported to correlate with ID [5, 6].

Globally, more than two billion people are at risk of IDD, of which 32% are school children, and the total goiter rate is estimated to be 15.8% [7]. One-third of the global community is living in areas where natural sources of iodine are low [8]. Furthermore, the highest prevalence of ID is documented in Africa (42%) [7]. Of the African countries, the largest burden is found in Ethiopia, which reported 39.9% of iodine-deficient children [9]. According to the Ethiopian demography and health survey (EDHS, 2016), only 15% of children aged 6 to 59 months living in households that use adequate iodized salt [10]. School-age children are considered as an appropriate target group for determining iodine deficiency due to their susceptibility to ID, easy accessibility as a study group, and representativeness of their community society as a whole [11].

Urinary iodine concentration in a population usually defines endemic iodine deficiency. Besides, it has been used as ID indicators [12]. This study aimed to determine the level of urinary iodine concentration and its associated factors among school-aged children in Dessie city, Amhara region, Northeast Ethiopia.

## Materials and methods

### Study design and period

A school based cross-sectional study was employed to determine the level of urine iodine concentrations among school children aged 6 to 14 years in Dessie city, Amhara region, Ethiopia from April to June 2019.

### Study area

The study was conducted in Dessie city administration in Amhara regional state, Northeast Ethiopia which has 18 urban and 8 rural districts. Based on the 2007 Central Statistical Agency of Ethiopia, this city has an estimated total population of 151,094 of whom 72,891 are men and 78,203 are women [13]. Dessie city is located at an altitude of 2470 m above sea level and has a subtropical highland climate.

### Source population and study subjects

A total of 57 primary schools have been found in Dessie city, of which 45 are governmental and 12 are private schools. In 2018, an estimated 38,068 (18,900 male and 19,168 females) school children aged 6 to 14 were found in the city. All children aged 6–14 years residing in Dessie city were the source population and randomly selected children from the schools were the study subjects. Children with known cases of iodine deficiency, diabetes, cardiovascular disease, and who took any vitamin and minerals before 7 days of sample collection, and who are unable to communicate or respond at the time of collection were excluded from the study.

### Sample size and sampling techniques

The sample size was determined using the single population proportion formula by considering the following assumptions: 5% margin of error, 95% confidence level, and iodine deficiency prevalence of 50%. After adding a 10% non-response rate and 1.5 design effect, a sample size of 634 was obtained. Firstly, from 57 schools 12 were selected randomly, then a list of all eligible children aged 6–14 years was taken from the respective schools for sampling frame. Secondly, proportion to size allocation was made to determine the required sample size from each school and a systematic random sampling technique was applied to select the required numbers of children from each school. Children who were absent on the date of data collection were substituted by the next student in the sampling frame.

### Data collection

Data were collected using interviewer-administered questionnaires from mothers/caregivers. The tool/questionnaire was first prepared in the English language and then translated to the Amharic version, which is the resident’s mother tongue and the national working language. The questionnaire was developed from World Health Organization resources and similar studies. Ten milliliter median spot urine sample was collected using a clean sterile screw cup container, stored at -20^0^c, and transported by maintaining its cold chain to Ethiopian Public Health Institute for analysis. Sandell–Kolthoff reaction on microplates followed by colorimetry was used for iodine.

### Data quality control

To ensure data quality, data collectors and supervisors were trained and regular supervision and follow-up were made by supervisors. Safety procedures and standard operating procedures were followed during laboratory testing. Before the actual data collection, the questioner was pre-tested on 5% of the total sample size of the respondents from Kombolcha town school children.

### Data analysis

The data was checked for completeness, cleaned, coded, and entered into Epi-data version 3.2 then, exported to the SPSS version 21 for analysis. Descriptive frequencies and summary statistics were used to describe the characteristics of study participants. Results were presented using tables and figures. Logistic regression was used to evaluate the presence and degree of association between each independent and outcome variable. Odds ratio with 95% confidence level and *P*-value < 0.05 was considered statistically significant.

#### Operational definitions

- Iodine deficiency: Urinary iodine measurement is < 99.9 μg/L. When people do not have enough iodine, they cannot make enough thyroid hormone. These consequences such as Goiter, Hypothyroidism, Cretinism, Reproductive failure, Childhood mortality, and Socioeconomic retardation (ICCIDJ, 1990)
- Adequate iodine: Urinary iodine measurement between 100 to 299.9 μg/L
- Excess iodine: Urinary iodine measurement > 300 μg/L

## Results

### Socio-demographic characteristics of children and their parents/guardians

A total of 634 school children aged 6 to 14 years were enrolled in the study with a median age of 12 years (±SD = 2.0). The majority of study participants, 358(56.5%), were females and more than half were between 12 to 14 years of age. Most of the children’s mothers, 79.2%, were married, and based on occupational status 44.5% were government employees. Forty-four, 6.9%, of the participants had a family history of goiter (Table 1).

**Table 1:** Socio-demographic characteristics of school children aged 6 to 14 years and their parents/guardians, Dessie, Amhara region, Ethiopia, 2019

Characteristics	Category	Frequency	Percent
Sex of children	Male	276	43.5
	Female	358	56.5
Age of children	6–8	76	12.0
	9–11	221	34.9
	12–14	337	53.2
Marital status of mothers/guardians	Married	502	79.2
	Single	35	5.5
	Divorced	73	11.5
	Widowed	24	3.8
Educational status of mother/ guardians	Unable to read &write	151	23.8
	Read-write	174	27.4
	Primary education	222	35.0
	Secondary and above	87	13.7
Family size	< 5	514	81.1
	≥ 5	120	18.9
Family history of goiter	Yes	44	6.9
	No	590	93.1

### Utilization and awareness of iodized salt

A substantial proportion, 88.3%, of households reported the use of iodized salt in their diet on a regular basis. Regarding the awareness of iodized salt, about 44.1% of the mothers/guardians heard about iodized salt and the main source of information was radio/TV, 43.8%, followed by health professionals, 25%. Only 3.3% of the households had storage condition of salts in an open container while nearly 91% of the mothers/guardians reported storage conditions far from the fire and 96.2% away from sunlight exposure. Although the majority of respondents had an acceptable level of awareness on the importance of iodized salt, about 89.6% of them add salt at the late end and at the end of cooking (Table 2).

**Table 2:** Utilization and awareness of iodized salt among parents/guardians of school children aged 6 to 14 years Dessie, Amhara region, Ethiopia, 2019

Variable		Frequency	Percent
Did you hear about iodized salt?	Yes	456	71.9
	No	178	28.1
From what source you heard about iodized salt?	Health professionals	159	25.1
	Radio/TV	278	43.8
	Internet	2	.3
	Others sources	16	2.5
Do you use iodized salt?	Yes	560	88.3
	No	74	11.7
At what frequency you use iodized salt?	Occasional	112	17.7
	Mostly	83	13.1
	Always	366	57.7
Type of container used to store salt	Open	21	3.3
	Closed	613	96.7
Sunlight exposure during storage of salt	Yes	24	3.8
	No	610	96.2
Storage of salt and distance from fire?	Near to fire	56	8.8
	Away from fire	576	90.9
	Both	2	0.3
During cooking, at what time do you apply the salt?	Early	7	1.1
	Middle	59	9.3
	Late end	526	83.0
	After	42	6.6

### Consumptions of goitrogenic food

As Table 3 shows, goitrogenic foods were consumed with different frequencies and cabbage was the highest at 87.5% with 44.2% of participants consumed more than once per week. Milk scored second at 78.4% consumption and 38.6% of participants reported consumption of more than one per week which is followed by sorghum and millet was the least consumed.

**Table 3:** Consumption of goitrogenic foods among school children aged 6 to 14 years, Dessie, Amhara region, Ethiopia, 2019

Type of food	Category	Frequency	Percent
Sorghum consumption	Yes	476	75.1
	No	158	24.9
Frequency of Sorghum consumption(*N* = 476)	> 1/week	210	33.1
	< 1/week	266	42.0
Millet consumption	Yes	159	25.1
	No	475	74.9
Frequency of millet consumption(*N* = 159)	> 1/week	69	10.9
	< 1/week	90	14.2
Cabbage consumption	Yes	555	87.5
	No	79	12.5
Frequency of cabbage consumption (*N* = 555)	> 1/week	280	44.2
	< 1/week	275	43.4
Milk consumption	Yes	497	78.4
	No	137	21.6
Frequency of milk consumption (*N* = 497)	> 1/week	245	38.6
	< 1/week	251	39.6

### Urinary iodine level

In the present study, the median urinary iodine level was 158.5 μg/L ranging from 5.1 μg/L to 528.8 μg/L. The overall proportion of iodine deficiency was 18.6%, of which severe, mild, and moderate deficiencies accounted for 7.4, 3.8, and 7.4% respectively. Adequate iodine level was observed in 80% of the study subjects while 11.8% had excess urine iodine levels. (Table 4).

**Table 4:** Urinary iodine level among school children aged 6 to 14 years, Dessie, Amhara region, Ethiopia, 2019

Urinary Iodine Status	Reference Range (μg/L)	Frequency	Percent	Cum. percent
Severe deficiency	< 20	47	7.4	7.4
Mild deficiency	20–49.9	24	3.8	11.2
Moderate deficiency	50–99.9	47	7.4	18.6
Adequate	(100–299.9)	441	69.6	88.2
Excess	> 300	75	11.8	100
Total		634	100	

### Factors associated with iodine deficiency

**Fig. 1:**
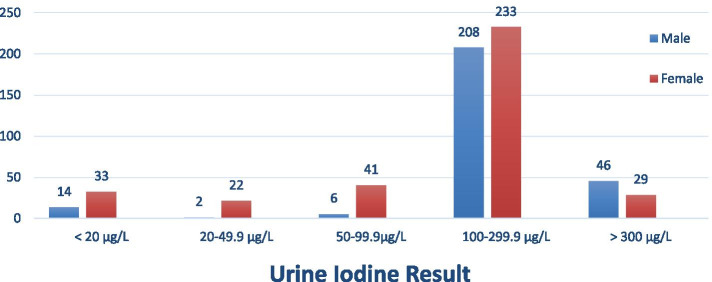
Distribution of urine iodine level by sex among school children aged 6 to 14 years, Dessie, Amhara region, Ethiopia, 2019

From the data in Fig. 1 below, it is apparent that the proportion of any type of iodine deficiency was higher in females than males. In line with this, excess iodine level was observed among males than females (Fig. 1). No significant differences were observed between urinary iodine level and age. However, children with age between 9 to 11 years had 49 (25.7%) urine iodine deficiency followed by those aged 12 to 14 with 63 (20.9%), (Table 5).

**Table 5:** Binary and multivariable logistic regression analysis of iodine deficiency among school children aged 6 to 14 years, Dessie, Amhara region, Ethiopia, 2019.

Variable name		Iodine deficiency status		COR(95% CI)	p-value	AOR (95% CI)	P-value
		Iodine deficient	Iodine adequate				
Sex	Male	22	208	1		1	
	Female	96	233	3.89 (2.36–6.42)	< 0.001	4.75(2.65–8.52)	< 0.001
Age in years	6–8	6	60	1		1	
	9–11	49	142	3.45(1.4–8.48)	0.007	3.47(1.35–8.87)	0.009
	12–14	63	239	2.63(1.08–6.38)	0.03	2.63(1.05–6.60)	0.038
Educational status of the mother	Unable to read write	20	113	1		1	
	Able to read write	45	112	2.27(1.26–4.08)	0.06	2.15(1.16–3.98)	0.01
	Primary education	32	161	1.12(0.61–2.06)	0.70	1.01(0.53–1.92)	0.95
	Secondary education and above	21	55	2.15(1.08–4.31)	0.02	2.05(0.98–4.28)	0.05
Do you hear about iodized salt	Yes	90	310	1		1	
	No	28	131	0.73(0.46–1.17)	0.20	0.65(0.39–1.09)	0.10
At what place do you store salt	Open	12	7	1		1	
	Closed	429	111	0.44 (.17–1.15)		0.27(0.09–0.77)	0.01

Bivariate logistic regression analysis showed the age of children, sex of children, mother education, dietary intake of iodized salt, being aware of iodized salt, frequent use of iodized salt, and storage condition of iodized salt were identified as factors associated with iodine deficiency with *p*-value less than 20%. However, as depicted in Table 5, in a multivariable logistic regression analysis, the sex of the child and the storage condition of iodized salt were the only independent variables significantly associated with iodine deficiency.

The results revealed that the sex of the child was significantly associated with deficiency of iodine, as a result being female was 4.75 times more likely to be iodine deficient than males (AOR = 4.75; 95% CI: 2.648, 8.52). The storage condition of iodized salt was another factor significantly associated with iodine deficiency. Storage of iodized salt with the closed container at household level was 0.27 reduced odds of iodine deficiency as compared with open container storage (AOR = 0.27; 95% Cl: 0.09–0.77) (Table 5).

## Discussion

The median urinary iodine level in this study was 158.5 μg/L ranging from 5.1 μg/L to 528.8 μg/L which is higher than a study in Bahir Dar, Amhara region Ethiopia and Aira district, west Ethiopia that showed a median urinary iodine level of 58.8 mg/L (12.89 mg/L to 564.5 mg/L) [14] and 70.5 μg/l respectively [15]. Another study in Anchar district, Eastern Ethiopia, reported a median urinary iodine concentration of 146 μg/L which is comparable with the present study. On the other hand study in Shebedino woreda, southern Ethiopia the higher median urinary concentration, 518 (327 to 704 μg/L) was seen this increment might be because the study was done after the 5 years implementation of the national salt iodization program [16].

The present study showed that the overall iodine deficiency among school children aged 6 to 14 years was 18.6% indicating high prevalence. This finding is similar to several sub-national studies in Ethiopia that showed iodine deficiency as a public health problem in the country [8, 17–20]. This might be due to the fact that many parts of the country are known for their mountainous topography and the top layer of the soil has been eroded for decades leading to the leaching away of nutrients including iodine. However, a sharp regional difference was observed including and urban and rural areas. Moreover, a much severe iodine deficiency, 64%, in Burie and Womberma Districts, West Gojjam, Ethiopia was seen which might be due to excessive use of goitrogenic chemicals such as Dichlorodiphenyltrichloroethane (DDT) and pesticides [4] .

In our finding, the sex of the child was significantly associated with iodine deficiency. As a result, being female was 4.75 times more likely to be iodine deficient. This finding is consistent with studies conducted in other parts of Ethiopia [21]. This could be justified by the higher vulnerability of females to iodine deficiency than males due to early puberty, the inhibitory effect of estrogen on iodine uptake, and also its goitrogenic effect by increasing thyroid follicular proliferation.

Age is an important factor associated with iodine deficiency, as age increases the iodine requirement also increases to support rapid growth. In our finding, high proportion of iodine deficiency was seen among the age group between 9 to 14 years. This age is the period of early adolescence where iodine requirement increases due to physiological and hormonal changes. To compensate for this requirement the body utilizes more iodine which is supported by various studies [18, 22].

Some foods have an effect on thyroid function by inhibiting the synthesis of thyroid hormone having a similar effect as iodine deficiency and are termed goitrogenic. In the current study, cabbage was the highest consumed, 87.5%, goitrogenic food which is higher than previous studies. This might be because of frequent consumption due to its cheap cost and easily availability [21, 23].

Awareness of parents/guardians about iodized salt and regular utilization is an important component of iodine deficiency elimination programs. Despite the fact that iodized salt is used in 88.3% of households, the salt’s iodine content is significantly influenced by its storage conditions. Storage in closed containers at household level was 0.27 reduced odds of iodine-deficient as compared with open container storage (AOR =0.27; 95% Cl: 0.09, 0.77) which could be due to the volatile nature of iodine. In our finding, about 97% of the respondents were aware of the reduction in iodine content of iodized salt if not stored in closed containers in line with previous findings [17], that recommends packaging with a strong moisture barrier, such as low-density polyethylene containers, and in most cases, salt can be made with reasonably stable iodine content for at least 6 months [24]. The most frequently mentioned sources of information on iodized salt in this study was radio/television,43.8%, which is consistent with a study in other parts of Ethiopia [17, 25]. The second source of information was trained health educators, reflecting the focus of a nationwide program to promote the consumption of iodized salt using these routes.

## Conclusions

In our finding, iodine deficiency of the school children aged 6 to 14 was 18.6% indicating a high prevalence with severe deficiency of 7.4% which might indicate application of iodine supplementation for more vulnerable groups. A substantial proportion, 88.3%, of households, was using iodized salt regularly. However, a quite few proportions of households and parents/guardians exhibit limitations in proper storage of iodized salt such as the use of the closed container, storing salts away from fire and heat. Moreover, the awareness of the mothers/guardians was low as only 44.1% of them have heard about iodized salt, suggesting intensifying efforts to increase knowledge and practice towards utilization of iodized. The high proportion of iodine deficiency was observed among females than males and it increases as the age increases. An implication of these findings is that both sex and age should be taken into account in iodine deficiency control programs.

## Data Availability

All the datasets during and/or analyzed during the current study are available from the corresponding author on reasonable request.

## Abbreviations

DHS: Demographic and Health Survey Report
ETB: Ethiopian Birr
FT4: Free Fraction of Thyroxin
ID: Iodine Deficiency
IDD: Iodine Deficiency Disorder
SAC: School-Age Children (6–12 years)
TSH: Thyroid Simulating Hormone
UI: Urinary Iodine
UIL: Urine Iodine level
WHO: World Health Organization

## Declarations

We declare this is our original work.
